# Focusing Conservation Efforts on Ecosystem Service Supply May Increase Vulnerability of Socio-Ecological Systems

**DOI:** 10.1371/journal.pone.0155019

**Published:** 2016-05-11

**Authors:** Pedro Laterra, Paula Barral, Alejandra Carmona, Laura Nahuelhual

**Affiliations:** 1 Consejo Nacional de Investigaciones Científicas y Técnicas (CONICET)–Fundación Bariloche, Av. Bustillo 9500, 8400, San Carlos de Bariloche, Argentina; 2 Instituto Nacional de Tecnología Agropecuaria (INTA, EEA Balcarce), CC 276,7620, Buenos Aires, Argentina; 3 Center for Climate and Resilience Research (CR2), Santiago, Chile; 4 Instituto de Economía Agraria, Universidad Austral de Chile, Casilla 567, Valdivia, Chile; 5 Centro de Investigación: Dinámica de Ecosistemas Marinos de Altas Latitudes (IDEAL), Santiago, Chile; UC Santa Cruz Department of Ecology and Evolutionary Biology, UNITED STATES

## Abstract

Growing concern about the loss of ecosystem services (ES) promotes their spatial representation as a key tool for the internalization of the ES framework into land use policies. Paradoxically, mapping approaches meant to inform policy decisions focus on the magnitude and spatial distribution of the biophysical supply of ES, largely ignoring the social mechanisms by which these services influence human wellbeing. If social mechanisms affecting ES demand, enhancing it or reducing it, are taken more into account, then policies are more effective. By developing and applying a new mapping routine to two distinct socio-ecological systems, we show a strong spatial uncoupling between ES supply and socio-ecological vulnerability to the loss of ES, under scenarios of land use and cover change. Public policies based on ES supply might not only fail at detecting priority conservation areas for the wellbeing of human societies, but may also increase their vulnerability by neglecting areas of currently low, but highly valued ES supply.

## Introduction

Growing international concern about the loss of ecosystem services (ES), mainstreams the ES approach into public policy, strengthening the link between human wellbeing and ecosystem integrity [1, 2]. Spatially explicit quantification of ES supply (also provision or flow), is widely recognized as a key tool in this endeavor [3–7]. In turn, advances in scientific knowledge and the emergence of GIS-based mapping tools set the groundwork for the exponential growth of diverse mapping methods [8–11]. Presently, these methods range from simple shape algebra to complex process-based models; and from ad-hoc procedures developed for specific case studies to standardized mapping routines [12, 13]. Such a wide array of methods and resulting map outcomes, while valuable from a scientific standpoint, might confound decision makers when different procedures are proposed for similar objectives (e.g. land-use planning or ES payment design), or when distinct planning goals are analyzed with similar methods [11].

Types of ES-based maps are not equally suitable for different purposes. For example, final maps of current ES supply can properly inform the design of payments for ES, However, informing long-term agriculture or forestry expansion policies requires trade-off analysis, and ES maps conflict under different exposure to land use and cover change (LUCC) scenarios [14]. Also important to consider is the susceptibility of ES supply to said exposure and their adaptive capacities to withstand it.

Integration of ecological and social dimensions into socio-ecological vulnerability maps due to loss of ES flows is an important step for the internalization of the ES approach into public policy and governance of natural capital. The latter is especially true for countries such as those in the Latin American region, where accelerated LUCC fosters social inequalities [15]. This paper illustrates how uncritical use of ES supply maps, may not contribute to the desired link between human wellbeing and ecosystem integrity, and could lead to the opposite outcome. We performed two case studies in Argentina and Chile in order to compare resulting conservation priorities according to ES supply versus socio-ecological vulnerability maps, using an improved version of ECOSER [16, 17], a GIS-based mapping tool.

## Methods

A detailed description of our methods is provided in the supporting information (S1 and S2 Files). Main aspects of ECOSER procedures, case study information and data analysis are summarized in the following sections.

### From ecosystem functions to vulnerability of the socio-ecological system

**Fig 1:**
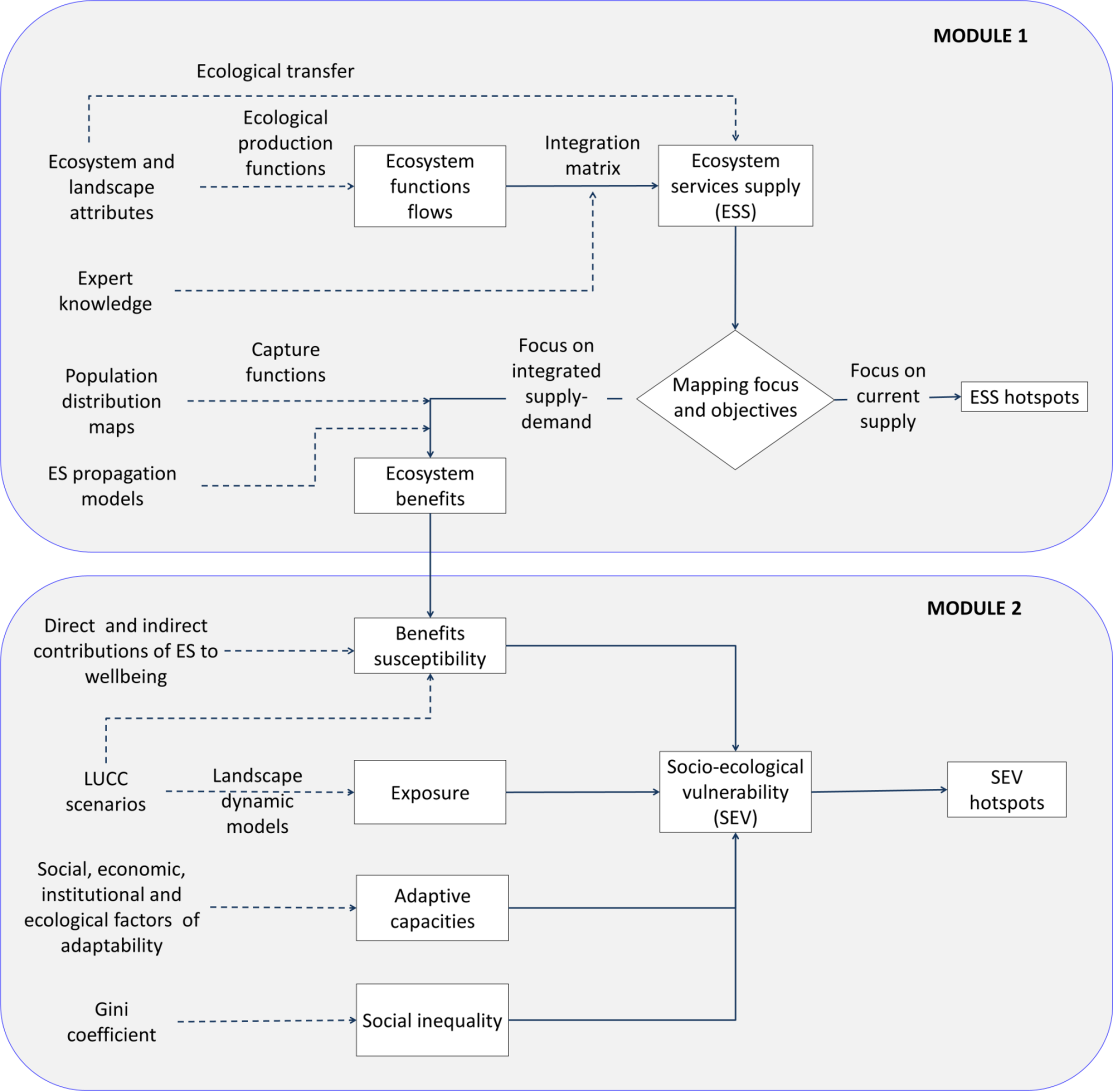
ECOSER 2.0 flow chart. Main components (rectangular boxes), calculus routes (full arrows), and data inputs (broken arrows) for the separate assessment and mapping of different types of ES. Arrows pointing to hotspots of ES supply and socio-ecological vulnerability represent the overlapping of different ES maps. Selection procedures of target ES are out of the scope of ECOSER. LUCC: land use and cover change.

By extending the generally accepted definition of system vulnerability, for the present study we understand socio-ecological vulnerability as the degree to which a socio-ecological system is susceptible to, or incapable of facing the adverse effects of a specific pulse or pressure (anthropic or natural perturbation) [18, 19]. This may adversely compromise the capture and flow of ES, as well as the social distribution of their benefits. Therefore, socio-ecological vulnerability depends on a variety of mutual interactions and feedback mechanisms between the social (e.g. social actors and governance) and ecological subsystems (e.g. ecological processes and functions). While socio-ecological vulnerability cannot be directly observed, it was inferred and quantified using ECOSER, which is based on the integration of three complementary conceptual frameworks: a) the socio-ecological systems approach [20–22], which embraces b) the ES cascade model [23] (Module 1 of ECOSER), and c) the vulnerability to ES loss [24] (Module 2 of ECOSER) (Fig 1).

Module 1 leads to the quantification, integration, and mapping of different components of the ES cascade, from ecosystem and landscape attributes to ES benefits, through three basic steps. First, ecosystem functions or intermediate ES [25], are calculated from ecosystem and landscape attributes, by applying ecological production functions [8] or ecological transfer procedures (see S1 File for details). Second, ecosystem functions are weighted by expert judgment and linearly combined in the calculus of ES flows. In the final step, ecosystem benefits are derived from ES supply after affecting them by capture functions reflecting the capacity of transforming ES supply into benefits (contribution to wellbeing).

Because of data limitations, we reduced capture functions to simple capture coefficients, i.e. the fraction of total population in the study area which has access to the ES supply. Therefore, in our model, we do not consider actual demand for the ES, and the capture coefficient and benefits represent maximum or potential figures which can be adjusted using real demand or value estimations. Capture of indirect benefits from ES and contributions to wellbeing from economic activities sustained by ES supply [26], are included in Module 2 within the susceptibility calculus as a multiplier factor reflecting relative influence of direct benefits from the *i*-ES type on local indirect benefits (see S1 File for details).

Module 2 is linked to Module 1 by the expected marginal changes in benefits according to a chosen scenario of LUCC. It also leads to socio-ecological vulnerability through the difference between benefit susceptibility to LUCC and the adaptive capacity of the socio-ecological system to withstand, cope with or adapt to those changes. These factors are all affected by exposure represented by the probability of LUCC occurrence, as follows:

$${SEV}_{ij}=(a*E)*[\;I*(\left(b*S_{ij})-(c*{C_{i}}^{\left({cb}_{i}\right)}\right))]$$ (1)

where *SEV* is the socio-ecological vulnerability due to benefit loss from i-ES type in the pixel *j*, *E* is exposure to LUCC, *S_ij_* is susceptibility to benefit loss in the pixel *j* for the *i*-ES type, *C_i_* is the adaptive capacity of the socio-ecological system for the *i*-ES type, *cb_i_* represents the relative contribution of the *i*-ES type to the overall wellbeing, and *a*, *b* and *c* are parameters that represent specific weights for each component of socio-ecological vulnerability according to the initial state of the socio-ecological system (see adopted values in S1 File Table C). *I* is a coefficient of inequality that increases the calculated socio-ecological vulnerability when social asymmetry in susceptibility and/or adaptive capacity increases.

Adaptive capacity, the ability of a socio-ecological system to moderate potential damages, to take advantage of opportunities, or to cope with the consequences of LUCC, is specific to the type of ES and depends on a combination of ecological, social, economic, and institutional factors (S1 File Table D).

Ecosystem service supply and maps of socio-ecological vulnerability are the main outputs provided by ECOSER, but additional products can also be obtained such as maps of benefits, susceptibility, and adaptive capacity. The ECOSER flowchart (Fig 1) was separately applied to different ES, which were only combined in the final step as multiple hotspot maps.

### Case studies description and sources of data

In order to illustrate the differences between ES supply maps and socio-ecological vulnerability, and highlight their implications for decision making, two case studies are described and discussed. The study areas correspond to Ancud County in Chiloé Island, Chile, and Mar Chiquita Basin, Buenos Aires, Argentina. Twelve ecosystem function indicators, comprising process models and indices, were integrated into three ES types for the Ancud area (i.e. availability of clean surface water, recreation opportunities, and potential firewood production) and into two ES types for the Mar Chiquita area (i.e. availability of clean groundwater and flood regulation). These ES types were selected for illustrative purposes and do not represent all the relevant ES for the study cases, nor the entire set of ES that currently compose ECOSER. Elements of the integration matrixes were obtained from expert consultations comprising 15 and 7 experts from Chile and Argentina, respectively, and are presented as supporting information (S2 File).

Businesses as usual scenarios were used for estimation probabilities for both study sites. Transition probabilities were obtained from 1999–2007 period for Ancud, and from 1999–2007 and 1999–2011 periods for Mar Chiquita, based on land cover maps.

Availability of secondary data for Module 2 was limited for the two case studies. Therefore, relative contributions of a given *i*-ES type to overall wellbeing were adopted from the default option of ECOSER (based on MEA [2]; see S1 File). Ratios of indirect beneficiaries to direct beneficiaries were used as proxies of local indirect benefits derived from ES supply of different ES types, according to expert judgment on economic activities supporting local employment and incomes. Also, because of data limitations, a mix of general and ES type-specific indicators were used as economic and institutional factors of adaptive capacity to loss of supply form different ES types (see S2 File). Exposure, benefit susceptibility and adaptive capacity were evenly weighted (or unweighted) in the calculation of socio-ecological vulnerability for both study cases. See S2 File for more details on criteria, assumptions, parameters and data sources used for the assessment of ES supply and socio-ecological vulnerability.

Specific factors to represent adaptive capacities were not easy to find for Mar Chiquita, so we opted for more general adaptive capacity indicators. In the Ancud case, different indicators were available and were used as economic and institutional factors of adaptive capacity to loss of different ES types (see S2 File).

### Data analysis

Landscape planning in general, and conservation planning in particular, both usually target specific areas of high ecological value or highly valued attributes for a better allocation of monetary resources. We rely on the concept of hotspots, defined here as pixels within the upper 10% and 20% percentiles of the frequency distributions of ES supply and socio-ecological vulnerability for each ES type (hereafter, 10% or 20% hotspots of ES supply, and 10% or 20% hotspots of socio-ecological vulnerability, respectively).

Ecosystem service supply and socio-ecological vulnerability hotspots were calculated and mapped for each ES type in both study cases. This allowed for inter and cross comparative analysis of ES supply and socio-ecological vulnerability for the five selected service types. In order to simplify the visual comparison, multiple hotspot maps for the complete set of ES types were produced for each study case, by calculating the number of hotspots of ES supply or the number of socio-ecological vulnerability hotspots of different ES types by pixel.

Redundancy or congruence between the highest scoring areas in ES supply and socio-ecological vulnerability were separately evaluated for each service by comparing their hotspot maps. Comparisons were performed using the Jaccard similarity coefficient (J), that is, the size of the intersection, measured as common hotspot pixels between maps, divided by the size of the union of the sample sets, measured as the total hotspot pixels of both maps. The upper 10% and 20% percentile maps were compared, but in order to simplify graphical representation, only maps of 20% multiple hotspots of ES supply and socio-ecological vulnerability are shown. Main variation patterns of socio-ecological vulnerability components (exposure, susceptibility and adaptive capacity) were explored, taking into account their correlation structure, and using Principal Component Analysis. The relationship between principal components and unweighted socio-ecological vulnerability was described using Pearson correlation coefficients. Statistical significance was not assessed because of the obvious co-linearity between socio-ecological vulnerability and its components.

## Results

### Ancud case

**Fig 2:**
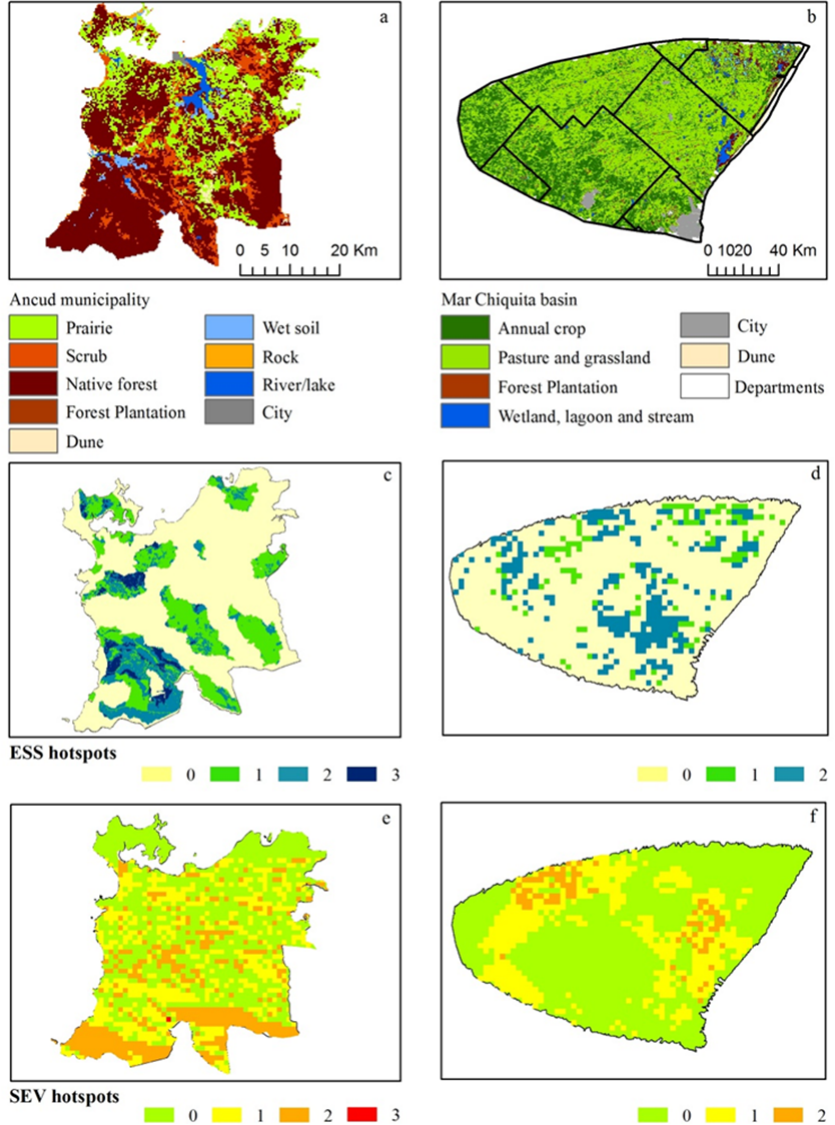
Maps of Ancud and Mar Chiquita study cases. Land-use and land-cover (a and b), multiple hotspots of ES (c and d), and multiple hotspots of socio-ecological vulnerability (SEV) (e and f) within the study cases (Ancud and Mar Chiquita, respectively). Three, two, one, or zero categories in maps c and d correspond to the occurrence of three, two, one, or none hotspots of ES supply (ESS), respectively. Three, two, one, or zero categories in maps c and d correspond to the occurrence of three, two, one or none hotspot of socio-ecological vulnerability, respectively. Internal lines in map b represent limits of counties.

The 20% hotspots of recreation opportunities are associated to Chiloé National Park and its buffer zone, and to the banks of the county’s main river and wetland (Pudeto wetland), located in the southwest study area (Fig 2C). All of these areas are mostly covered by native old growth and secondary forests. Additional hotspot areas for recreation opportunities are located to the east, where exotic forest plantations of *Eucalyptus* spp. and *Pinus* spp. (mostly for timber and chips) are spatially associated with secondary forests. In contrast, hotspots of firewood provision and aboveground water coincide with native forests concentrated in the south of the county within the buffer zone of Chiloé National Park. These are areas with low population density and low residential development.

Spatial distribution of ES supply hotspots show poor similitude between all paired comparisons of ES types: recreation opportunities versus firewood (J = 18%), aboveground water provision versus firewood (J = 27%), and aboveground water provision versus recreation opportunities (J = 19%). The spatial overlap of these three different hotspots represents a negligible portion (2.3%) of the county's area (Fig 2).

**Fig 3:**
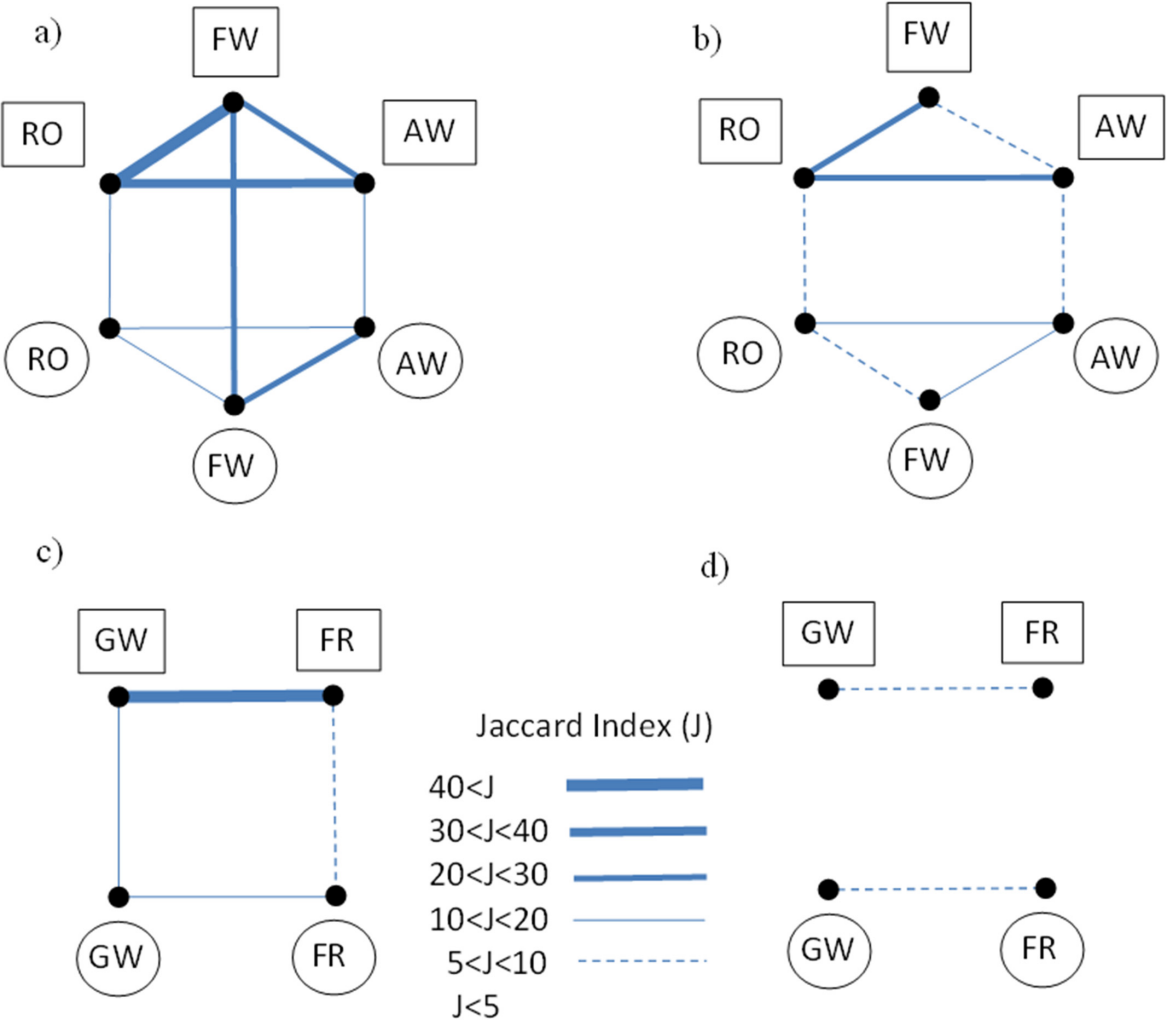
ES supply and socio-ecological vulnerability similarities. Degrees of similarity among hotspots of ES supply (circles) and hotspots of socio-ecological vulnerability (rectangles) maps, for the upper 20 (a and c) and 10 (b and d) percentiles for Ancud (a and b) and Mar Chiquita (c and d). Different line thickness indicates different degrees of similarity according to Jaccard index values. AW: aboveground water provision, GW: groundwater provision, FR: flood regulation, RO: recreation opportunities, FW: firewood provision. Similarity between ES supply and socio-ecological vulnerability of different ES types was not calculated.

Similarity of spatial distribution between pairs of socio-ecological vulnerability hotspots from different ES was even lower than that observed for hotspots of ES supply (Fig 2), and represented 3.8%, 3.8% and 3.6% of the Ancud area for recreation opportunities versus firewood provision, aboveground water provision versus firewood provision, and aboveground water provision versus recreation opportunities, respectively. However, spatial coincidence of 20% hotspots of socio-ecological vulnerability for the three ES types was higher (3.8% of the Ancud area) than the area of spatial overlap for hotspots of ES supply (Fig 2). According to their similarity coefficients, spatial overlap between ES supply and socio-ecological vulnerability hotspots was very low for all ES types (Fig 3).

### Mar Chiquita case

The 20% hotspots of the two ES types had a dissimilar spatial distribution (J = 14%, Fig 3). The highest portion of these intersecting hotspot cells was located in non-cultivated areas of the Flooding Pampa region (Fig 2A and 2D). In these areas, erosion control, retention of excess rainfall and groundwater protection by perennial pastures and grasslands, plus the relatively high quality and groundwater depth, represented high contribution to both groundwater provision and flood regulation (S1 File Table B).

Similarity of spatial distribution of socio-ecological vulnerability hotspots between the two mapped ES (J = 42%, Fig 3), was higher than that observed between ES supply hotspots. Therefore, hotspot maps of socio-ecological vulnerability for different ES types partly masked the observed differences between hotspots of ES supply. According to their spatial distribution on maps (Fig 2) and to their similarity coefficients (Fig 3), spatial congruence of ES supply- and socio-ecological vulnerability hotspots was very low for all ES types.

### Exposure, susceptibility and adaptive capacity: main variation patterns

According to Principal Component Analysis, the relative influence of exposure, susceptibility and adaptive capacity on socio-ecological vulnerability varied among ES types within the same case study. This interpretation is based on: a) the first principal axis or factors were highly or moderately correlated with socio-ecological vulnerability for all ES types in both case studies; and b) the elements or loadings of each component of socio-ecological vulnerability within the first factors reflect their relative contribution to variation in socio-ecological vulnerability (Table 1). For example, the main variation in socio-ecological vulnerability due to potential loss of firewood in Ancud is explained by the co-variation of the three main components of socio-ecological vulnerability, which share high loadings with the same sign within the factor F1 (Table 1). Areas with relatively high exposure have also comparatively high susceptibility and low adaptive capacity. It is important to remark that adaptive capacity enters to the socio-ecological vulnerability equation with negative sign.

**Table 1:** Principal Component Analysis of socio-ecological vulnerability.

	Ancud case						Mar Chiquita case			
	Firewood provision		Recreation opportunities		Aboveground water provision		Groundwater provision		Flood regulation	
	Factor 1	Factor 2	Factor 1	Factor 2	Factor 2	Factor 1	Factor 2	Factor 2	Factor 1	Factor 2
Exposure	-0.74	-0.44	0.82	0.13	0.81	0.17	0.95	-0.05	0.94	-0.13
Susceptibility	-0.81	-0.09	-0.82	0.17	-0.81	0.16	0.06	1.00	0.36	0.93
Adaptive capacity	-0.47	0.86	0.04	0.98	0.01	0.98	-0.95	0.01	-0.92	0.23
Eigenvalue	1.42	0.94	1.35	1.02	1.31	1.02	1.8	1.00	1.87	2.8
% Total variance	47.00	31.00	45.00	34.00	44.00	34.00	60.10	33.30	62.00	31.00
r	-0.97	0.17	0.88	0.09	0.76	0.19	-0.46	-0.16	-0.24	0.09

Elements of each factor are the loadings of exposure, susceptibility and adaptive capacity within the first two factors of each principal component analysis. Pearson correlation 16 coefficients (r) were calculated between factor scores and socio-ecological vulnerability at the pixel level.

Only exposure and susceptibility accounted for variations in socio-ecological vulnerability due to loss of recreation opportunities. Exposure and susceptibility also explained variation in socio-ecological vulnerability because of losses in aboveground water provision in Ancud. The latter can be explained since adaptive capacity variation was absorbed by the second factors which were not correlated with socio-ecological vulnerability (Table 1). In contrast with socio-ecological vulnerability, due to firewood loss, main patterns of variation in socio-ecological vulnerability for recreation opportunities and for above water provision were determined by opposite trends between exposure and susceptibility. Hence socio-ecological vulnerability of areas with relatively high exposure were compensated by low susceptibility scores, and vice versa. For example, areas with high exposure to loss of recreation opportunities did not show high scores of socio-ecological vulnerability because of their low potential capture and hence low benefit and susceptibility scores. Finally, as occurs for the groundwater provision and flood regulation cases in Mar Chiquita, adaptive capacity overrides the influence of exposure on socio-ecological vulnerability; areas with relatively low exposure scores have a tendency to show high scores of socio-ecological vulnerability because of their comparatively poor adaptive capacity.

## Discussion

### Distribution of ES supply does not reflect the vulnerability of socio-ecological systems to ES loss

Map comparisons in terms of spatial distribution of the upper 20% and 10% hotspots, reveal two main patterns for both study sites. Firstly, maps of socio-ecological vulnerability do not show spatial correspondence with ES supply maps of the same service type. Secondly, socio-ecological vulnerability maps of different ES types are more similar among themselves than ES supply maps of the same service type. In the first case, partial correspondence is expected given the mathematical dependence of susceptibility on ES supply. However, observed differences between hotspots of ES supply and socio-ecological vulnerability, both for individual ES and bundles, reflect the importance of other supply and socio-ecological vulnerability components apart from ES flows in determining supply and socio-ecological vulnerability maps, as observed for both case studies. This also reflects different resolution scales of available data. Sharp differences between hotspots of ES supply and hotspots of socio-ecological vulnerability are observed for the Mar Chiquita case (Figs 2 and 3), because of the high contribution of exposure and adaptive capacity to variation in socio-ecological vulnerability (Table 1). Also, grain of adaptive capacity data (i.e. county level) is coarser than grain of ES flow data (3x3km cells) (Fig 2B, 2D and 2F).

As previously reported [27], components of socio-ecological vulnerability for different ES types are not independent, but describe patterns of spatial co-variation (Table 1). This result suggests that appropriate decisions for reducing socio-ecological vulnerability cannot emerge from unconnected land use and social policies. For example, reduction of socio-ecological vulnerability due to loss of potential firewood provision in Ancud does not only require reduction in exposure of native forest to replacement by other land covers, but also reduction in susceptibility to that loss. This can be accomplished by improving the efficiency of access to ES benefits and/or by improving the social distribution of benefits. Another way is to improve firewood production in transformed landscapes (Table 1). In contrast, according to the main variation pattern of socio-ecological vulnerability due to potential loss of groundwater provision in Mar Chiquita, reductions in socio-ecological vulnerability are more likely to be achieved through the reduction of exposure and the improvement of adaptive capacities than through the reduction of susceptibility to benefits loss.

### Socio-ecological vulnerability outperforms ES supply as land-use planning criteria

Our results support the basic idea that socio-ecological vulnerability is a spatially heterogeneous phenomena, and thus calls for spatially-explicit public policies. Hotspot maps of socio-ecological vulnerability may support the identification of areas where decisions may prevent the highest costs in terms of wellbeing loss.

Since planning based on ES supply or socio-ecological vulnerability maps can lead to different conservation priorities, the logical—yet overlooked—question emerges of what the best land use planning criteria should be, under extant ecological, social and political contexts. In this regard, we sustain that ES supply maps represent a first stage on a stairway of information that might be complementarily used for effective decision making, where vulnerability evaluation lies at the top of the stairway. Yet, vulnerability assessments that integrate ES as key elements to link ecosystem conservation to wellbeing are still very scarce [27–31].

As a decision making tool, maps of ES supply hotspots are straightforward measures of current or projected ES richness and concentration, regardless of the probability of ES flows to generate wellbeing for local and distant populations. On the other hand, socio-ecological vulnerability maps can highlight areas that may not have such richness and concentration, but where ES flows are highly valued by people. Furthermore, socio-ecological vulnerability maps account for social adaptive capacities to cope with ES loss under selected scenarios of land use change. In summary, vulnerability might reflect more effectively the interactions between ecological and social systems, thus providing fuller indicators for land use planning.

**Fig 4:**
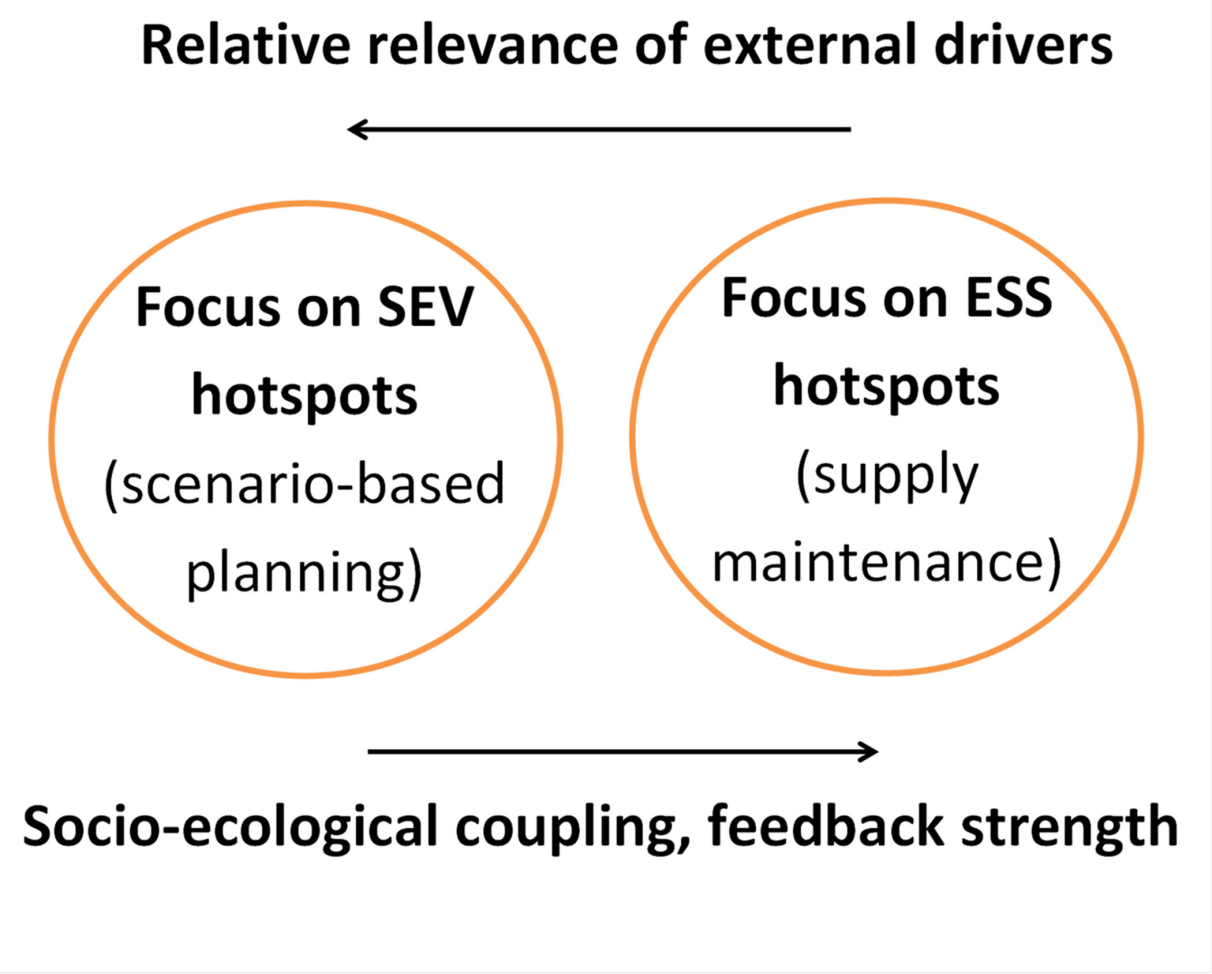
Focus on socio-ecological vulnerability (SEV) versus on ecosystem services supply (ESS), two approaches for pursuing human wellbeing through nature conservation. Differences in relevance of external drivers and degree of socio-ecological coupling call for different strategies of nature conservation.

Following the inquiry about the relative suitability of ES supply versus socio-ecological vulnerability maps, it is also convenient to consider some relevant differences between the conceptual frameworks supporting both kinds of maps. The ES approach [1, 32] is aimed at promoting the sustainability of socio-ecological systems through the maintenance of ES supply by efficient local governance based on close connections between human wellbeing and the integrity of surrounding natural capital (coupled condition). While the ES approach promotes the construction of ES supply maps as key final products for supporting human-nature feedbacks [7, 10–12], strongly coupled conditions are very rare in real world socio-ecological systems. Poor coupling between local nature and human wellbeing due to low perception of ES loss, technological substitutes, growing influence of external drivers on local natural capital, and/or poor communication between local stakeholders and decision makers, are all important challenges to the ES approach. To aid the overcoming of these challenges, the ES approach can be merged with vulnerability frameworks. Therefore, along gradients of local human-nature uncoupling, it is possible to envisage two contrasting foci for pursuing human wellbeing through nature conservation: a) maintenance of ES supply under low influence of external drivers on natural capital and wellbeing and efficient feedbacks between local nature and society, and b) reduction of socio-ecological vulnerability to ES loss under the opposite conditions (Fig 4).

Hotspots of socio-ecological vulnerability may not only result from high exposure to LUCC, but from high susceptibility of ES benefits to that exposure, and/or from low capacity of the social system to adapt to ES benefits losses. Such losses can be due for example to the absence of ES substitutes and social networks. In this context, mapping socio-ecological vulnerability can provide evidence not only of where a socio-ecological system is vulnerable, but also why it is vulnerable. A socio-ecological system can be vulnerable because it is highly susceptible and lacks adaptive capacity, or because it is highly exposed and adaptive capacities are insufficient. Different types of vulnerability thereby call for different policy interventions.

Demand and access to ES as well as the adaptive capacities to ES loss are neither homogeneously nor normally distributed within society [33, 34] so their mean or general value for a given socio-ecological system may be masking socio-ecological system dynamics. Moreover, disproportionate (non-additive) influences of asymmetric distributions in susceptibility and adaptive capacities may arise from their non-random combinations [35]. Unfortunately, statistical distributions of susceptibility and adaptive capacities and their associations are hard to obtain for real socio-ecological systems, where aggregated data for administrative units is a common situation. Therefore, as an attempt to include this asymmetric phenomena in the calculus of socio-ecological vulnerability, we introduce a coefficient of inequity that increases socio-ecological vulnerability values when social asymmetry in susceptibility and adaptive capacity increases.

Our results support the idea that conservation focused on areas of highest ES supply, or hotspot areas, are not necessarily the best policy target for promoting effective contribution of ES to wellbeing. Moreover, because of spatial uncoupling between hotspots of ES supply and socio-ecological vulnerability, focus on ES supply may not only fail in detecting priority conservation areas for the wellbeing of human societies but also may increase their vulnerability by neglecting areas of currently low, yet highly valued ES supply.

### Next steps

Similar to other spatially-explicit vulnerability assessments and tools, ECOSER outputs are contingent upon considerations such as the choice of underlying conceptual frameworks, the variables selected to represent such concepts (e.g. capture of benefits), selected LUCC scenarios, aggregation of datasets, spatial resolution of data and analysis, and variable weighting. Additionally, there are significant underlying assumptions in any effort to identify aggregate socio-ecological vulnerability such as for example, the linear relation between exposure, susceptibility and adaptive capacity. ECOSER builds on ecosystem functions, ES and captured benefits as pillar objects to represent vulnerability, objects which are complex in their own. Still, there might be important variables that are left out or insufficiently represented within our protocol. For these reasons map outcomes presented here must be interpreted and used with caution. Nevertheless, despite the caveats noted above, we argue that given the data available, and given the difficulty in modelling future socio-ecological conditions, these maps provide a useful first step in assessing broad scale socio-ecological vulnerability.

Bridging ES and vulnerability frameworks renders several challenges to scientific research. In order to increase reliability for decision making, mapping socio-ecological vulnerability with ECOSER, or other analogous tools, calls for further improvements such as: a) expert validation of the relationships between ecosystem functions and ES for different socio-ecological contexts, b) consideration of ES thresholds within ecological production functions, c) adjustment of ES supply modeling by sustainability criteria (e.g. carrying capacity in case of recreation opportunities; ecological flows in case of water provision), d) cautious consideration and distinction of demand for and effective use (capture) of ES by society, e) revision of scales assigned to ordinal variables; for example relative contribution of ES types to the overall wellbeing and factors used for the calculus adaptive capacity in order to avoid influences of scaling arbitrariness [36] on socio-ecological vulnerability scores, f) consideration of interactive influences among adaptive specific factors, and g) incorporation of uncertainty analysis.

The demand for, and the capture of ES supply are core concepts for assessing the susceptibility of a socio-ecological system to ES loss. In this study (and in the employed version of ECOSER), it was assumed that ES demand was completely reflected by ES capture, which in turn was approximated by the relative access (e.g. distance functions) to the ES supply. Simplistic assessment and mapping of demand were made through overlaying population and ES supply maps [37, 38]. Improved procedures are required in order to provide a more realistic representation of ES demand. It is necessary to disentangle ES capture from demand, considering different linking patterns between ES supply and beneficiaries according to spatial propagation, rivalry and excludability of particular ES types [39]. Other demand-related aspects which are included within ECOSER procedures, such as: a) the inclusion of indirect (economic) benefits into susceptibility computation (see S1 File for details), b) inclusion of socio-economic inequality, correcting the susceptibility and adaptive capacity, c) consideration of demand (and supply) thresholds, d) reciprocal supply and demand interactions, e) interactive and not merely additive influences of demand by different ES types, and f) variation of ES demands among social groups are commonly neglected by previous proposals for the assessment and mapping of ES. Sensitivity analysis of socio-ecological vulnerability maps with different descriptors of inequality and indirect benefits may reveal the relevance of these neglected aspects.

Identification and quantification of uncertainties are overlooked in nearly all published assessments of ES [40], and this study is not an exception. Multiple uncertainty sources affect nearly every step of different ecological models, indices, and vulnerability elements included in assessment of socio-ecological vulnerability [41], making their uncertainty analysis a difficult, but necessary task. Therefore, how uncertainties of ES supply and socio-ecological vulnerability maps can be hierarchized, estimated, described and communicated to decision makers should be addressed by forthcoming studies.

## Conclusions

Results of this paper offer relevant directions and tool improvements for ecosystem service researchers, practitioners and policymakers. Our main conclusion is that prioritized conservation areas based only on current ES flows poorly account for the spatially and temporally changing contribution of ES to human wellbeing.

In these times of globalization, when landscape dynamics, ES supply and social distribution of ES benefits are increasingly affected by remote drivers, main components of socio-ecological vulnerability cannot be neglected. Decision makers should be aware that public policies, instruments, and land-use decisions based on current maps of ES supply will probably fail in detecting priority conservation areas. Awareness of this dynamic will increase the possibility of the wellbeing of human societies under changing landscapes. Moreover, maps of current ES supply cannot inform about socio-economic factors that limit social adaptive capacities to withstand expected ES losses.

Coordinated decisions are not facilitated by unconnected thematic maps within institutionally limited contexts [42]. Moving from policies and decisions based on the supply or a demand side versus the integrated supply and demand of ES requires appropriate frameworks and tools. Despite previous attempts at bridging ES with vulnerability and socio-ecological systems [43], mapping and assessment of socio-ecological vulnerability are still in their infancy.

## Supporting Information

S1 File ECOSER fundaments and description. (PDF)

S2 File Case studies and data sources. (PDF)

S3 File Datasets. (XLS)
