# Supplementary material for: Focusing Conservation Efforts on Ecosystem Service Supply May Increase Vulnerability of Socio-Ecological Systems
Source: PLoS One. 2016 May 11;11(5):e0155019. doi: 10.1371/journal.pone.0155019 (PMC4864224; doi:10.1371/journal.pone.0155019)
Supplement: S1 File — (PDF) [file pone.0155019.s001.pdf]

S1File. ECOSER 2.0 fundaments and description

Supporting Information of the paper "Focusing conservation efforts on ecosystem services supply may increase the vulnerability of socio-ecological systems" by Laterra, P, Barral, P, Carmona, A, Nahuelhual, L (PLOS ONE, 2016).

General overview

ECOSER 2.0 (hereafter ECOSER) is a GIS modeling framework aimed to support local to regional land-use planning and sustainable development policies, where the current supply (or flow) of relevant ecosystem services (ES) types from different landscape units is not necessarily the unique or the best criteria. In such cases, maps showing the hotspots of socio-ecological vulnerability due to loss of ES supply under different scenarios of land-use and cover change (LUCC) may be considered as better criteria for definition of public policies than single maps of ES supply. Therefore, ECOSER provides maps of ES supply and socio-ecological vulnerability through the integration of three complementary conceptual frameworks: a) the socio-ecological system, which embraces b) the ES cascade [1] (module 1), and c) the vulnerability analysis (module 2) (see Fig. 1 in the main text).

The ecological subsystem within a socio-ecological system is defined by the natural capital, and associated functions and services. The ES types most readily incorporated into the social system are the goods (provisioning services) that are directly harvested and used by human beings (e.g. crop, timber, water). Additionally, there are regulating services such as weather and flood regulation that augment the spatial scale of social-ecological interactions from individual stands to landscapes; and cultural services that provide a sense of place and identity, aesthetic or spiritual benefits, and opportunities for recreation opportunities and tourism. The social subsystem, however, is defined by economic, political, and cultural characteristics that constitute a society and define human existence at a particular place.

The flow chart of ECOSER presented in Fig. 1 (main text) show the final outputs, main procedures and the required data inputs within the two basic modules: the assessment and mapping of ecosystem service benefits from ecosystem and landscape attributes (Module 1), and the assessment and mapping of socio-ecological vulnerability from those benefits, LUCC scenarios, and social, economic and institutional information (Module 2). Conceptual and procedure details within each step of ECOSER flowchart are presented along the following sections.

Scores of socio-ecological vulnerability at any mapping unit depend on ecological and social properties which vary at different scales because of the very nature of their supporting processes and/or because of data availability, so scale mismatches can be expected within and between the ecological and social dimensions. For example, mapping of socio-ecological vulnerability requires a minimum set of proxies of social, economic and institutional variables which are frequently

aggregated at administrative scales with lower resolution than the available biophysical data. Therefore, maps of socio-ecological vulnerability obtained by ECOSER do not necessarily reflect some real extent of a socio-ecological system, but arbitrary boundaries within which heterogeneity of socio-ecological vulnerability and its components are evaluated and represented. Since SEV maps may be sensible to the scale of analysis, scale needs to be carefully selected by the ECOSER users, and limitations to policy making from arbitrary selections need to be evaluated and declared.

The design of this ECOSER version was not only intended for the integration of ecological and social dimensions of ES into socio-ecological vulnerability, but also for attending to some highly desirable but less frequent properties: a) context sensitivity of ES supply and delivery, b) indicators generality, c) and continuous improvement, as described below.

Information at any pixel of ECOSER maps not only depends on local properties of these pixels, but according to the ES type, it may also depends on properties of other surrounding pixels affecting its capacity to sustain a given ES supply, and/or its capacity to deliver part of that supply to the enjoyers. For example, the ecosystem function of filtering of runoff by a given pixel covered by a riparian ecosystem depends on the filtering efficiency at that pixel, as well as on the sediment load in the incoming runoff (which is approached through the land-use in the collector area which drains to the pixel of interest). One resulting ecosystem benefit, water purification, is mapped on the same pixel after the ES supply is affected by the amount of people that is potentially benefited by it, depending on the spatial propagation of the ES. Like runoff filtering, other important ecosystem functions can be more properly called landscape functions, since they depend on lateral fluxes of matter and/or energy, and cannot be properly evaluated without taking into account particular landscape properties. Similarly, landscape benefits are represented on the pixel where the ES is supplied, but according to the portion of the landscape where it is potentially enjoyed and according the people living within it.

Aiming at land-use planning as the main objective of mapping, selection of production functions must follow suitability, reliability, and relevance criteria. Suitability of proxies (ecological production functions like indices and process models) vary according to the information and knowledge levels available for a given study site. ECOSER is not defined by a particular set of proxies of ES supply but by the general procedures introduced in this overview and detailed below. Here we selected a particular set of proxies because of their suitability for the study cases (S1 File Table A), however ECOSER is flexible enough for the incorporation of alternative proxies. The present application of ECOSER to the study cases is based on preliminary integration matrices for their respective ecoregions. However, given the exponential growth of relevant knowledge and synthesis, and possible interest for application in diverse landscapes and socio-ecological systems, continuous addition, improvement or adaptation of ecological production functions and integration matrices is in the core of the suitability and reliance of this tool for future applications in different contexts. Since this objective is out of reach of a single research group, we designed ECOSER for promoting the interdisciplinary collaboration, and a web site offering opportunities to upload, download and comment complementary or alternative indices, process models, and matrices [2].

S1 File Table A. Ecological production and ecological transfer functions selected for the assessment of study cases.

Ecosystem function	Ecological production/transfer function	Ref.
Soil organic carbon storage	$SOC_i = SOC_{ref} \times F_{lu_i} \times F_{mg_i} \times F_{a_i}$<br>*$SOC_i$*: estimated soil organic carbon stock in pixel i (Mg C)<br>*SOC_{ref}*: initial soil organic carbon stock (under reference condition, pristine or semi-pristine condition, Mg C)<br>*F_{lui}*: change factor related to the type of land-use/land cover in pixel i.<br>*F_{mgi}*: change factor related to labors practices in pixel i.<br>*F_{ai}*: change factor related to different levels of carbon input to the soil.	[3]
Biomass carbon storage	$BCS_i = B_{LULC_i}$<br>*BCS_i*: carbon storage in biomass in pixel I (TonC/pixel)<br>*$B_{LULC:}$* biomass carbon storage values assigned to each land-use/land cover.	[3]
Erosion control	$EC_i = RUSLE_{max_i} - RUSLE_i$<br>*$EC_i$*: erosion control in pixel i (Ton/pixel)<br>*$RUSLE_{max}$*: Universal Soil Loss Equation parameterized for bare soil in pixel i.<br>*$RUSLE_i$*: Universal Soil Loss Equation parameterized for cover class in pixel i.	[4]
Soil fertility	$SF_i = PI_i$<br>*SF_i* : soil fertility in pixel i<br>*PI_i* : productivity index in pixel i	[5]
Retention of rainfall excess by vegetation	$ERV_i = \left(P - \frac{(P - 0.2IE)^2}{P + 0.8IE}\right) * storm_{days} \qquad IE = \frac{25400}{(254 + NC)}$<br>*$ERV_i$* : retention of rainfall excess by vegetation in pixel i (mm).<br>*P*: total precipitation of a single-event rainfall (mm)<br>*$storm_{days}$*: number of days of storm per year.<br>*IE*: rainwater retained by the ecosystem before runoff occurs.<br>*NC*: curve number, a coefficient taking a value within the range of 0–100, related to land-use, permeability, soil moisture before rain, and topography.	[6, 7]
Retention of rainfall excess by wetlands	$ERW_i = WA_i * TWI_i \qquad TWI_i = ln\left(\frac{a_i}{\tan \beta_i}\right)$<br>*$ERW_i$*: retention of rainfall excess by wetlands in pixel i.<br>*WA_i*: wetland area<br>*TWI*: Topographical Wetness Index<br>*A_i*: upslope contributing area in pixel i.<br>*$Tan\beta_i$*: slope in pixel i.	[8]

Aquifer protection by vegetation	$AP_i = 1 - (ID_{rel_i} * P_i)$<br>$AP_i$: aquifer protection by vegetation in pixel i.<br>ID_{reli}: DRASTIC index in pixel i.<br>P_i: protection factor of cover type in the pixel i.	[9]
Sediments and pollutants retention in wetlands	$N_{ret} = 7.56 * NT_{in}^{0.49} * A^{0.51}$ $NT_{in} = NT_0 * e^{-k*t}$<br>$P_{ret} = PT_{in}^{0.96} * 0.34$ $PT_{in} = PT_0 * e^{-k*t}$<br>$N_{ret}$: total nitrogen (NT) retained by the wetland area (kg/pixel/year)<br>NT_{in}: total nitrogen entering the wetland area (kg/pixel/year)<br>NT_0: initial nitrogen mass that is exported from each pixel.<br>*A*: wetland area<br>P_{ret}: total phosphorous (PT) retained by the wetland area (kg/pixel/year)<br>PT_{in}: is the amount of PT entering the wetland area.<br>PT_0: initial phosphorus mass that is exported from each pixel.<br>*k*: decay coefficient.<br>*t*: travel time.	[10 - 13]
Sediments and pollutants retention in riparian vegetation strips [1]	$SR_{RVSi} = C_i * SRE_i$ $SRE_i = 53.35 + 235 * RA$<br>$C_i = erosion_i * SDR$ $SDR = 0.4724 * A^{-0.125}$<br>$SR_{RVS}$: sediment retention in the i-riparian vegetation strip (RVS)<br>SRE_i: efficiency of sediment retention rank in the i-RVS<br>*RA*: ratio area, is the ratio between the area of the riparian vegetation strip area and source area.<br>C_i: pollutants loading rank.<br>$erosion_i$: potential erosion, calculated with RUSLE equation.<br>*SDR*: sediment delivery ratio<br>*A*: watershed area	[13 - 17]
Landscape structure	$SI_i = a * type_i + b * struc_i + c * soil_i + d * patch\_size_i$<br>*a, b, c, d*: specific weights from expert opinions<br>$type_i$: forest type in pixel i<br>$struc_i$: forest structure (old growth, secondary, etc) in pixel i<br>$soil_i$: type of soil in pixel i<br>$patch\_size_i$: size of patch to which pixel i belongs	-
Biomass production	$V_i = G_i + HDOM_i - N_i$<br>V_{SIi}: specific formula of densometric volume.<br>V_i : densometric volumen per pixel i.<br>G_i: basal area in pixel i.<br>$HDOM_i$: dominant stand high in pixel i.<br>N_i: number of trees in pixel i.	-

	$H_i = a * typeslope_i + b * Soil_i + c * dist_i$	-
Habitat production for species	a, b, c, d: specific weight from expert opinions<br>$typeslope_i$: range of slopes preferred by target species i<br>$soil_i$: range of soil types preferred by target species i<br>$dist_i$: distance to dispersal pathways that depends on target species i	

Relevance of production functions clearly depends on the relative importance or value of the ES for stakeholders, and not merely for the academic or technical sectors. Therefore, we emphasize the need to subordinate the selection of ES to the final objectives of the assessment. Methods to reveal social or economic values of ES are widely discussed and they are out of the ECOSER's scope, but a guide for the preliminary selection of ES types on basis to expert knowledge can be found in the ECOSER website [2].

From ecosystem and landscape attributes to ecosystem functions

This framework is currently integrated by a set of 12 ecosystem functions (or intermediate ecosystem services [2]: 1) soil organic carbon storage, 2) biomass carbon storage, 3) erosion control, 4) soil productivity, 5) retention of rainfall excess by vegetation, 6) retention of rainfall excess by wetlands, 7) aquifer protection by vegetation, 8) sediments and pollutants retention in wetlands, 9) sediments and pollutants retention in riparian vegetation strips, 10) landscape structure, 11) biomass production, and 12) habitat production for species. Ecological production functions for each ecosystem function were adopted or adapted from the bibliography, and modeled as toolbox scripts in ArcGIS environment (S1 File Table A). Proxies for this set of ecosystem functions were selected according to their theoretical consistency and the availability of required data, but we are aware that the same ecosystem functions can be approached by different proxies. The proposal, analysis, and selection of alternative proxies are in the basis of the evolving character that we are intending for this framework, through learning processes along collaborative work.

In addition to the ecological production functions, ECOSER flowchart also includes an alternative pathway from ecosystem and landscape attributes to ES supply, the "ecological transfer" procedures, consisting in the assignation of ES supply values obtained from other studies o from expert knowledge to homogeneous cover classes (e.g. biomes or vegetation units, among others). Ecological transfer procedures may include the correction of ES supply values by simple local information like terrain slope or patch size for diminishing extrapolation errors, but they are essentially land cover based methods with little or no support of causal relationships. Like other spatial value transfer procedures, ecological transfer may be used as an alternative for ecological production functions when knowledge and/or information about ecosystem functions is lacking, as

well as for ecosystem services which are more easily linked to site attributes than to ecosystem functions (e.g. cultural services).

How cultural services like recreation opportunities can be spatially defined and visualized continues to be a challenge in the ES literature [18, 19]. Since the assessment of recreation opportunities is rather subjective and value-leaden, supply of this ES type is related both to the observer values and ecosystem conditions [20, 21], which are not easily linked to ecosystem functions but to ecosystem or landscape attributes (Fig. 1). In the extreme, cultural services essentially depends on the interaction of ecological and historical factors (neglected by the moment by ECOSER procedures) that cannot be reduced to ecological functions [22].

From ecosystem functions to ecosystem services supply

Supply of the i-ES (ESS_i) (or provision of final ecosystem services [2]) are obtained from the pixel to pixel linear combination of j-ecosystem function maps, weighted by the relative contribution of each j-ecosystem function (EF_j) to the i-ecosystem service, as follows:

$$ESS_i = \sum_{i=1}^{n} b_{ij} * EF_j \qquad (1)$$

The b_{ij} factors (0-1 rank) are the elements of the N ES columns by R ecosystem function rows of the integration matrix (see for example the integration matrix for the study cases, S1 File Table B). Since different ecosystem functions have different units, they must be normalized into a 0-1 scale before their combination, using as references the maximum and minimum values of the ecosystem functions within the study area. Therefore, is worth to note that normalization for commensurability purpose constraints the aggregation and/or the comparison of maps were maximum and minimum references are not the same.

S1 File Table B. Integration matrixes for Valdivian Forest Region and Mar Chiquita basin.

	**Ecosystem services types**				
	Ancud			Mar Chiquita	
	AW	RO	FW	GW	FR
**Ecosystem functions**					
Soil organic carbon storage	0.3[a]	0.0	0.0	0.3	0.2
Biomass carbon storage	0.2	0.0	0.0	0.2	0.2
Erosion control	0.9	0.0	0.0	0.6	0.7
Soil fertility	0.1	0.0	0.4	0.2	0.2
Retention of rainfall excess by vegetation	0.7	0.0	0.0	0.6	1.0

Retention of rainfall excess by wetlands	0.7	0.0	0.0	0.6	1.0
Aquifer protection by vegetation	0.4	0.0	0.0	1.0	0.3
Sediments and pollutants retention in wetlands	1.0	0.0	0.0	0.8	0.3
Sediments and pollutants retention in riparian vegetation strips	1.0	0.0	0.0	0.8	0.3
Biomass production	0.0	0.0	1.0	0.0	0.0
Habitat production for species	0.0	0.8	0.0	0.0	0.0
**Ecosystem and landscape attributes**					
Landscape structure	0.0	0.8	0.7	0.0	0.0

[a] Assigned weights to the relative contribution of each function (rows) to each service type (columns), based on normalization of average scores from expert surveys. AW: aboveground water provision, RO: recreation opportunities, FW: firewood provision, GW: groundwater provision, CP: potential crop production

Elements of integration matrix are hard to obtain because most of scientific literature is focused on particular processes, dismissing how they interact to support ecosystem services. Ecosystem services (e.g. water regulation) are generally reduced to single processes or ecosystem functions (e.g. water infiltration) that are considered as proxies of the ES supply (e.g. water regulation), and they possibly vary according to the ecological context. However, the relative importance of different ecosystem functions supporting a particular ES flow can be approached from expert consultation for each ecoregion through different techniques, like expert valuations, Saaty matrixes, and neural networks. Elements of the integration matrix for study sites (S1 File Table B) were obtained from an *ad hoc* survey as a simple average of the scores from 4-6 local experts in ES type. Integration matrixes for different ecoregions of Latin America are provided by Weyland [23].

From ecosystem services supply to ecosystem services benefits

Within our conceptual framework, benefits are defined as the fractions of the ES supply which are captured or transformed into the wellbeing of local people. The calculation of benefits is made for each ES separately within each pixel were ES fluxes are generated, using an index based on the product among the ES flux and the number of people which is benefited, according to the following expression:

$$B_{i,j} = ESS_{i,j} * cap_{i,j} \qquad (2)$$

where $B_{i,j}$ is the benefit of the i-ES type in pixel j, $ESS_{i,j}$ is the flow or supply of the i-ES type in the pixel j (calculated as in Eq. 1) and $cap_{i,j}$ is the capture of the i-ES type in pixel j calculated with equation [3]. Since ES supply data and capture data are previously standardized to 0-100 and 0-1 ranks, respectively, the resulting benefit data has a [0, 100] rank.

In turn, the capture of the i-ES type in pixel j is calculated as,

$$cap_{i,j} = \frac{f(dist)}{TP} \qquad (3)$$

where $cap_{i,j}$ represents the fraction of total population in the area (TP) which is benefited by the supply of the i-ES (direct beneficiaries). Direct beneficiaries is the number of people living within the propagation area of the i-ES type, which in turn is delimited using a propagation function or a simple model of people benefited according to their distance to the provision area $f(dist)$.

From ecosystem services benefits to socio-ecological vulnerability

The conceptualization of socio-ecological vulnerability is a challenging one. It directs attention to such questions as: who and which components of a system are vulnerable to the multiple environmental and human changes underway, and where? How are these changes and their consequences attenuated or amplified by different human and environmental conditions? What can be done to reduce vulnerability to change? How more resilient and adaptive communities and societies can be built?

ECOSER is engaged in the assessment of vulnerability of the social-ecological system and it is therefore following the current trend of conducting integrative vulnerability research [24]. Socio-ecological vulnerability in ECOSER is defined as the degree to which a socio-ecological system is susceptible to or incapable of facing the adverse effects of a specific pulse or pressure (anthropic or natural perturbation) which compromises adversely the flow of ES, their benefits, and ultimately the satisfaction of human needs. Mapping socio-ecological vulnerability requires the integration of all the components of the cascade of ecosystem services framework [1] besides information from specific features of the social system (e.g. users, access to ES, institutions).

Main components of socio-ecological vulnerability are the same as those of general vulnerability framework: exposure, susceptibility, and adaptive capacities to withstand changes. Whereas under the general vulnerability framework elements exposure to a perturbation are particular elements like human beings, assets, ecosystems [25], ECOSER focuses on ecosystem services and related human wellbeing elements. Susceptibility indicates the condition or rate of response of the SES with regard to all perturbations and stresses within the system. In this case, susceptibility refers to the extent of changes in ecosystem services and related human wellbeing.

Susceptibility and adaptive capacities are neither homogeneously nor normally distributed within the society, so their mean or general value for a given socio-ecological system may be masking SES situations deserving particular concern. Moreover, disproportionate (non-aditive) influences of asymmetric distributions in susceptibility and adaptive capacities may be arisen by their non-random combinations [26]. Unfortunately, statistical distributions of susceptibility and adaptive capacities and their associations are hard to obtain for real socio-ecological systems, where aggregated data for administrative and/or cense units is the common situation. Therefore, as an attempt to include this asymmetric phenomena in the calculus of socio-ecological vulnerability, here we introduce a coefficient that increases the calculated socio-ecological vulnerability when social asymmetry in susceptibility and/or in adaptive capacity increases (coefficient of inequity, *I*).

Socio-ecological vulnerability is separately calculated for each i-SE in two moments, t_0 and t_1, before and after a given perturbation. The general mathematical expression of socio-ecological vulnerability (SEV) is the following:

$$SEV_{ij} = (a * E) * \left[I * \left((b * S_{ij}) - (c * C_i{}^{(cb_i)}) \right) \right] \qquad (4)$$

where SEV is the socio-ecological vulnerability due to benefit loss from *i-ES* type in the pixel j, E is exposure to LUCC, S_{ij} is susceptibility to benefit loss in the pixel *j* for the *i*-ES type, C_i is the adaptive capacity of the socio-ecological system for the *i*-ES type, cb_i represents the relative contribution of the *i*-ES type to the overall wellbeing, and *a*, *b* and *c* are parameters that represent specific weights for each component of socio-ecological vulnerability according to the initial state of the socio-ecological system (see adopted values in S1 File Table C). *I* is a coefficient of inequality that increases the calculated socio-ecological vulnerability when social asymmetry in susceptibility and/or in adaptive capacity increases. This framework presents four components of wellbeing: i) security, ii) health, iii) good social relationship, and iv) basics materials for "good living" and two types of relationship between ES and wellbeing components: i) intensity of the connection between ES and wellbeing and ii) potential of the ES to be mediated economic factors. Cbi was reported by MEA [27] according to expert knowledge as the mean intensity of the connection between ES and each wellbeing component using a three leveled scale: High (=1), Medium (=0,66) and Low (=0,33). Potential of ES to mediate economic factors was not taken into account since a similar included into the benefits estimations.

S1 File Table C. Normalized average contribution of ecosystem services to wellbeing (*cb*), according to MEA [27] framework

Ecosystem service type	Security	Material wellbeing	Health	Good social relations	Overall (average)
Provision	0.66	1	1	0.33	0.74
Regulation	1	1	1	0.33	0.83
Cultural	0.66	0.33	0.33	0.33	0.41

Inequality was approached by using the coefficient of inequity of Gini (G), re-scaled to the [1-2] rank by doing

$$I = 1 + G \qquad (5)$$

Exposure computation

As previously stated, socio-ecological vulnerability is calculated between two moments: the initial system's state and a future state that is represented by a projected scenario of land-use and cover (one that involves the potential loss of ES). ECOSER does not provide procedures for representing exposure explicitly, and hence a qualitative and quantitative understanding is needed of how the system would change in a future scenario in response to local or regional pressures. Exposure values are finally standardized to [0, 1] rank.

Susceptibility computation

This calculation requires understanding how benefits change in response to exposure. Susceptibility is calculated as:

$$S_i = cb_i * (B_{1i} - B_{2i}) * W_i \qquad (6)$$

where *i* is the ES type under evaluation, B_{1i} is the benefit arising from the *i*-ES type in t_0 and $B_{2\mathrm{i}}$ is the benefit arising from the same ES in t_1, cb_i the relative contribution of a given *i*-ES type to overall wellbeing standardized to [0-0.5]. By default, ECOSER calculates cb_i values as the weighted average of relative contribution of main ES types to each specific component of wellbeing provided by MEA [27], as shown in S1 File Table C. *Wi* is a multiplier factor which affects S_i according to the relative influence of direct benefits from the *i*-ES type on local indirect benefits (economic activities supporting local employment and incomes [28]). *Wi* can be guessed or estimated through different proxies like the number of indirect beneficiaries by direct beneficiary and production or income or employment multipliers from input-output models [29].

In the Ancud case, a mix of general and ES type-specific indicators was used as economic and institutional factors of adaptive capacity to loss of different ecosystem service types: For estimation of adaptive capacity of Ancud case, we used capacity factors: actual erodability (*Fecol*), the soils map of Región de Los Lagos and farms area [1] plus ES type specific substitution possibility and and ES type specific access to them (*Fecon*), and educational level (*Fsoc*) on basis to the National Population Census of 2002 [2].

For estimation of adaptive capacity of Mar Chiquita Ecosystem service type-specific factors of adaptive capacity were not easy to obtain as secondary data for the Mar Chiquita case, so we used some general adaptive capacity indicators (instead of ES type-specific indicators) case we used factor proxies that differed from those used for Ancud case due to limitations in data availability: number of job positions (*Fecon*), illiteracy level (*Fsoc*) and water entitlements (*Finst*) as provided by Ministry of Economy of Buenos Aires Province.

Adaptive capacities

Following the IPCC [5] definition for adaptive capacity, adaptive capacities (hereafter, C) represent the ability of a socio-ecological system to adjust to land use changes to moderate potential damages, to take advantage of opportunities, or to cope with the consequences. Adaptive capacities are ES type-specific and depend on the combination of four factor types, as follows:

$$C = Fecol + Fsoc + Fecon + Finst \qquad (7)$$

where *Fecol, Fsoc, Feco,* and *Finst* represent ecological, social, economic, and institutional factors, respectively, each factor resulting from the combination of factor proxies that can be plausibly associated to the adaptive capacity of the system (S1 File Table D). Since the relevance, availability and suitability of *F* proxies are context-dependent, they need to be necessarily selected or developed by ECOSER users. Indicators are weighted within factor types and factor types are weighted within *C*, which is finally standardized within the study site to 0-50.

S1 File Table D. Suggested variables for the calculus of social, economic, and institutional factors of adaptive capacity of socio-ecological systems.

Ecosystem service type	Security	Material wellbeing	Health	Good social relations	Overall (average)
Provision	0.66	1	1	0.33	0.74
Regulation	1	1	1	0.33	0.83
Cultural	0.66	0.33	0.33	0.33	0.41

[a] ES: ecosystem service: [b]ESS: ecosystem serices supply

Fecol is inversely related to main ecological factors reducing the recovering ability (resilience) of the ecological sub-system for a given disturbance: potential erodability (*E*), regeneration time of dominant species (*RT*) and a hyperbolic function of the distance to nearest remnant patches of the ecosystem, as a proxy of colonization opportunities (*D*):

$$Fecol = -[e\left(\frac{E_x - E_{min}}{E_{max} - E_{min}}\right) + r\left(\frac{RT_x - RT_{min}}{RT_{max} - RT_{min}}\right) + d\frac{D_x - D_{min}}{D_{max} - D_{min}}] \qquad (8)$$

where, *e*, *r* and *d* are weighting coefficients (0-1).

References

1. Haines-Young R, Potschin M. The links between biodiversity, ecosystem services and human well-being. In Raffaelli, D., Frid, C, editors. Ecosystem Ecology: A New Synthesis. Cambridge University Press, Cambride, UK; 2010.
2. Laterra, P, Nahuelhual, L., Barral, M. P. & Carmona, A. ECOSER. Collaborative Tool for the Assessment and Mapping of Ecosystem Services and Socio-ecological Vulnerability for Land Use Planning. URL: www.eco-ser.com.ar. At: www.eco-ser.com.ar Accessed September 2015.
3. IPCC (Intergovernmental Panel On Climate Change). Guidelines for national greenhouse gas inventories. 2006. At http://www.ipcc-tfi.iges.or.jp/meeting/pdfiles/Washington_Report.pdf Accessed July 2015.
4. Renar, KG, Foster GR, Weesies GA, McCool DK, Yoder DC. Predicting soil erosion by water: a guide to conservation planning with the revised universal soil loss equation (RUSLE), USDA Agriculture Handbook Number 703. Wash. DC; 1996.
5. Riquier J, Bramao D L, Cornet J. A new system of soil appraisal in terms of actual and potential productivity (first approximation). U. N. Food Agric. Organ. Soil Resour. Dev. Conserv Serv Land Water Dev. Div.; 1970.
6. Fu B, Wang Y K, Xu P, Yan K. Mapping the flood mitigation services of ecosystems - A case study in the Upper Yangtze River Basin. Ecol Eng. 2013;52: 238–246.
7. Cronshey, R. Urban hydrology for small watersheds. (US Dept. of Agriculture, Soil Conservation Service, Engineering Division). 1986. At https://tamug-ir.tdl.org/tamug-ir/handle/1969.3/24438 Accessed July 2015.
8. Laterra P, Orúe ME, Booman GC. Spatial complexity and ecosystem services in rural landscapes. Agric Ecosyst Environ. 2012;154: 56–67.
9. Aller L, Bennett T, Lehr J, Petty R. DRASTIC: A Standardized System for Evaluating Ground Water Pollution.157 (EPA/600/2-85/018); 1985.
10. Skop E, Sørensen P B. GIS-based modelling of solute fluxes at the catchment scale: a case study of the agricultural contribution to the riverine nitrogen loading in the Vejle Fjord catchment, Denmark. Ecol Model. 1998:106: 291–310.
11. Byström O. The nitrogen abatement cost in wetlands. Ecol Econ. 1998:26: 321–331.
12. Kadlec RH, Wallace S. Treatment wetlands. CRC Press; 1998.
13. Orúe ME, Booman GC, Cabria AF, Laterra P. Uso de la tierra, configuración del paisaje y el filtrado de sedimentos y nutrientes por humedales y vegetación ribereña. In Laterra P, Jobbágy, E, Paruelo JM, editors. Valoración de Servicios Ecosistémicos. Conceptos, herramientas y

aplicaciones para el ordenamiento territorial. Ediciones INTA. Buenos Aires, Argentina; 2011. pp. 359-390.

14. Muñoz-Carpena R, Parsons JE.VFSMOD-W. Vegetative Filter Strips Hydrology and Sediment Transport Modelling System. Model documentation & users manual version 2.x. 2003. At: http://abe.ufl.edu/carpena/files/pdf/software/vfsmod/VFSMOD_UsersManual_v6.pdf, Accessed: September 10, 2007.
15. Ouyang D, Bartholic J. Predicting sediment delivery ratio in Saginaw Bay watershed. In Proceedings of the 22nd National Association of Environmental Professionals Conference 1997. pp. 659–671.
16. Ouyang NL, Lu SL, Wu BF, Zhu JJ, Wang H. Wetland restoration potential estimation at the watershed scale -a case study in upstream of the Yongdinghe river. Wetl Sci 2012;1: 200–205.
17. Vanoni VA. Sediment Engineering: Manual and Report No. 54. N. Y. NY ASCE; 1975.
18. de Groot R , Alkemade R, Braat L, Hein L, Willemen L. Challenges in integrating the concept of ecosystem services and values in landscape planning, management and decision making. Ecol Complex. 2010;7: 260–272.
19. Maes J, Egoh B, Willemen L, Liquete C, Vihervaara, P, Schägner JP, et al. Mapping ecosystem services for policy support and decision making in the European Union. Ecos Serv. 2012;1: 31-39.
20. Hansen-Møller J. Natursyns model: a conceptual framework and method for analysing and comparing views of nature. Landsc Urban Plan. 2009;89: 65–74.
21. Kumar M, Kumar P. Valuation of the ecosystem services: a psycho-cultural perspective. Ecol Econ. 2008:64: 808–819.
22. Nahuelhual L, Carmona A, Laterra P, Barrena J, Aguayo, M (2014) A mapping approach to assess intangible cultural ecosystem services: The case of agriculture heritage in Southern Chile. Ecol Indic. 40: 90–101.
23. Weyland, F, Barral, P, Laterra, P. Vínculos entre funciones y servicios ecosistémicos: variaciones entre ecorregiones. Proceedings from the 4th International Congress on Ecosystem Services in the Neotropics Mar del Plata, Argentina, September 30 to October 3, 2015. 6 pp. www.geap.com.ar/cisen4/libro-resumenes/
24. McLaughlin P, Dietz T. Structure, agency and environment: Toward an integrated perspective on vulnerability. Glob Environ Change. 2008:18: 99–111.
25. Adger WN. Vulnerability. Glob Environ Change. 2006:16: 268–281.
26. Nowak, P, Bowen, S, Cabot, PE. Disproportionality as a framework for linking social and biophysical systems. Soc Nat Resour. 2006;19: 153–173.

27. MEA (Millennium Ecosystem Assessment). Ecosystems and human well-being: Synthesis World Resources Institute, Island Press, Washington DC, USA; 2005.

28. Daw T, Brown K, Rosendo S. Pomeroy, R. Applying the ecosystem services concept to poverty alleviation: the need to disaggregate human well-being. Environ Conserv. 2011;38: 370–379.

29. Miller RE, Blair PD. Input-output analysis: foundations and extensions. Cambridge University Press, Cambridge, UK; 2009.
