# Supplementary material for: Focusing Conservation Efforts on Ecosystem Service Supply May Increase Vulnerability of Socio-Ecological Systems
Source: PLoS One. 2016 May 11;11(5):e0155019. doi: 10.1371/journal.pone.0155019 (PMC4864224; doi:10.1371/journal.pone.0155019)
Supplement: S2 File — (PDF) [file pone.0155019.s002.pdf]

S2File. Case studies and data sources

Supporting Information of the paper "Focusing conservation efforts on ecosystem services supply may increase the vulnerability of socio-ecological systems" by Laterra, P, Barral, P, Carmona, A, Nahuelhual, L. (PLOS ONE, 2016).

Case studies

Ancud County in Chiloé Island, Chile, and Mar Chiquita basin, Buenos Aires province, Argentina were selected as case studies for comparing maps of ecosystem services (ES) supply and socio-ecological vulnerability maps upon contrasting ecological, social and economic conditions. All the ecosystem functions described in S1 Table A were integrated for deriving maps of different ES types: availability of clean surface water, opportunities of recreation opportunities, and potential firewood production for the Ancud case; availability of clean groundwater, and flood regulation for the Mar Chiquita case. This set of ES types was selected for illustrative purposes and it does not necessarily include all the relevant ES types for the study cases, or all the ES types currently considered by ECOSER.

The socio-ecological system of Ancud: general features, relevant ecosystem services and LUCC trends and scenarios

The municipality of Ancud is located in the northern part of Chiloé Island in the province of Chiloé in southern Chile. It covers a territory of 172,400 ha of which less than 1% is classified as urban. The remainder of the rural territory is comprised of 2854 farms, most of them under individual land tenure and with a farm area that ranges between 0.03 ha and 4658 ha [1, 2]. The study area can be considered marginal in terms of agricultural production, although agriculture continues to be a relevant source of rural income. These natural conditions have led to farming systems and rural livelihoods mostly oriented around self-sufficiency which combine a variety of activities such as small-scale fishing, timber logging, livestock breeding, and small-scale cultivation of cereals and potato. A large part of the study area is currently covered by relatively intact old-growth forest (53%, 55,686 ha) of which 11,776 ha are publicly protected by Chiloé National Park [3]. Among the human and natural processes occurring in the area, deforestation has been the most important in terms of its spatial extent [3, 4]. Forest degradation through logging also has been an important cause of forest loss [5, 6]. Although logging does not directly lead to loss of forest cover, it alters forest structure, composition, and function [4] and decreases the current and future economic value of the forest [7, 8]. Logging is basically practiced for firewood supply to both rural and urban populations, but firewood demand by rural population, where firewood is used not only for house heating but also for cooking and water heating, is higher than the demand from urban population [4, 7].

Another important driver of land change during the studied period was the change from forest and shrubland to exotic tree plantations (almost entirely Eucalyptus spp). Most of plantations replaced native vegetation (old-growth forest, secondary forest, and shrubland) and the rest consist in afforestation on marginal agricultural lands and flooded areas [3], [9]. These land-use changes also compromise the provision of another relevant ecosystem service like water provision and water flow regulation. Recent studies in southern Chile and Ancud in particular demonstrate how well-conserved native forest can improve water supply and how their replacement by exotic tree plantations can reduce water provision [10, 11].

Despite some adverse trends in land use and land cover, the natural heritage of Ancud continues to be valued and admired by people from around the world, who travel exclusively to experience Ancud’s numerous and diverse attractions. Among them are the Islets of Puñihuil and the northern part of the Chepu river, old growth temperate rain forests characterized by their high degree of endemism [12, 13], most of which are protected under Chiloé National Park, and other salient features such as whale feeding and breeding areas. Market studies conducted by SERNATUR in Chiloé establish that the most common activities practiced by tourists in Chiloé are wildlife observation (87%) and hiking (26.4%). According to other market surveys performed in 2008 for nature tourism in Chiloé, 36 % of visitors engage in nature-based activities such as hiking or agro-tourism and cultural activities.

All the selected ES types for this study case rely to different extent on the presence and condition (composition and structure) of native forests, and are affected by degradation from old-growth forest to two successional stages: secondary forest and shrublands. Therefore, a “business as usual” scenario was performed by calculating transition probabilities among the main forest stages between 1999 and 2007 and projecting it to 2015 on basis to classified satellite images.

The socio-ecological system of Mar Chiquita: general features, relevant ecosystem services and land-use change trends and scenarios

Mar Chiquita basin illustrate all the major regional land-uses of the Pampa ecoregion, including intensive agriculture, mixed agricultural-livestock systems and pure livestock systems in the lower fields [14]. The basin of Mar Chiquita coastal lagoon is located in the southeast of Buenos Aires province and within a half million hectares area, it includes portions of the Flooding Pampas and Southern Pampas of Argentina [15]. Eleven administrative units (departments) are wholly or partially included within the basin.

During the last 25 years, the Argentine Pampas region has experienced a continuous increase in the land area dedicated to annual crops in replacement of native grasslands and cultivated pastures. Since the early 1990s, structural economic changes fostered investment in technology which played a major role in land-use changes in the Pampas. Cattle breeding

(cow-calf systems) on native grasslands combined with cultivated pastures remained in non-agricultural lands but cattle fattening is progressively being expanded under intensive livestock practices [16]. All the mentioned changes in the production systems have resulted both in social costs, such as concentration of land ownership and migration of small farmers into cities [17], and in environmental costs, such as reductions in SE supply and biodiversity loss [16, 18, 19].

Argentine land-use planning experiences based on ES concept are very recent and have so far being focused on forest conservation, especially in the Northern provinces (Ley Nacional No. 26331) [20]. In the rest of the territory, there is neither land-use planning normative nor accepted political criteria for its application. For Mar Chiquita basin we adopted the spatially explicit business as usual scenario formulated according to historical trends of land-use related to socio-economic and biophysical driving factors [21, 22], from present to 2022, using Dyna- CLUE 2.0 [23].

Under current and projected scenarios, two ES types are particularly being affected: availability of clean groundwater and flood regulation. Groundwater is practically the unique source for drinking water and crops irrigation in the pampas, and its quality is being threatened by pollution from increasing agrochemicals applications [22, 24, 25]. Therefore, flooding is a recurrent disturbance affecting crop yields and cow-calf operations in the Flooding and Austral pampas, which are being probably enhanced upon the current transformation trend [26, 27].

Assumptions and data sources

Maps of the ecosystem functions detailed in S1 Table A were obtained using different information sources, so indicators of ES supply were evaluated at 100 m and 30 m resolution for the Ancud and Mar Chiquita cases, respectively, but maps were rescaled to 3x3km cells for Mar Chiquita and further analysis were performed at such scale.

For the Ancud case we employed a soil map with a resolution of 6.25 ha [28], land covers obtained from a Landsat satellite image (30mx30m) taken in [29], a digital elevation model and Google Earth images. Information for calculus of the Landscape Structure index was obtained from National Vegetation Resources Cadaster of Chile and its monitoring [29, 30], the soil base map and patch size were analyzed on basis to the National Vegetation Resources Cadastre [1]. Weighting of different components of Landscape structure was made through expert consult s. Ranges of slope, soil and dispersion distance for construction of the function indicator Habitat Production for Species, were obtained from Donoso et al. [31]. For the Mar Chiquita case, we employed a digital elevation model obtained from SRTM images (90x90m), a soil map 1:50000 [32] and land cover classification obtained from Landsat images (30x30m). Climate information was provided by the Argentina National Meteorological Service. Land cover maps were provided by the Catastro de Recursos Vegetacionales of Chile and the Laboratorio de Geomática, INTA Balcarce-FCA, UNMDP.

Population density for calculus of benefits within Mar Chiquita basin was mapped by extrapolation of population densities of cities and small towns using the inverse distance function.

In the Ancud case, a mix of general and ES type-specific indicators was used as economic and institutional factors of adaptive capacity to reduction in the supply of different ecosystem service types: actual erodability (*Fecol*), the soils map of Región de Los Lagos and farms area [1] plus ES-type specific substitution possibility and and ES-type specific access to them (*Fecon*), and educational level (*Fsoc*) on basis to the National Population Census of 2002 [2]. Ecosystem service type-specific factors of adaptive capacity were not easy to obtain as secondary data for the Mar Chiquita case, so we used some general adaptive capacity indicators (instead of ES type-specific indicators) that differed from those used for Ancud case: number of job positions (*Fecon*), illiteracy level (*Fsoc*) and water entitlements (*Finst*) as provided by Ministry of Economy (2015) of Buenos Aires Province.

Benefits of the selected ecosystem services were calculated on basis to assumptions detailed in S1 Table D. Inequity coefficients for Ancud and Mar Chiquita cases were calculated on basis to income distribution among properties, and the distribution of unmet basic needs among population, respectively.

References

1. CIREN (Centro de Información de Recursos Naturales), CORFO (Corporación de & Fomento). Digital Cartography of Rural Properties; 2009.
2. INE (Instituto Nacional de Estadísticas). VII Censo Nacional Agropecuario y Forestal; 2007. athttp://www.ine.cl/canales/chile_estadistico/censos_agropecuarios/censo_agropecuario_07.php> Accessed July 2015
3. Carmona A, Nahuelhual, L, Echeverría C, Báez A. Linking farming systems to landscape change: an empirical and spatially explicit study in southern Chile. Agric Ecosyst Environ. 2010;139: 40–50.
4. Echeverria C, Coomes D, Salas J, Rey-Benayas J M, Lara A, Newton A. Rapid deforestation and fragmentation of Chilean temperate forests. Biol Conserv. 2006;130: 481-494.
5. Armesto J J, Smith-Ramirez C, Carmona M R, Celis-Diez J L, Díaz I A, Gaxiola A, et al. Old-growth temperate rainforests of South America: Conservation, plant–animal interactions, and baseline biogeochemical processes. In Wirth et al. editors. Old-growth forests. Springer, Berlin. pp. 367–390.
6. Gómez-Lobo A. El consumo de leña en el sur de Chile:? Por qué nos debe preocupar y qué se puede hacer? Rev Ambiente Desarro. 2005;21: 43–47.
7. Reyes R (2000) Caracterización de los sistemas de producción y comercialización de leña para la ciudad de Puerto Montt, X Región. Thesis Santiago Univ. Chile.
8. Reyes R. Leña: Una oportunidad para la conservación de los bosques templados del sur de Chile. Rev Bosque Nativo. 2005;37: 16–25.
9. Marín SL, Nahuelhual L, Echeverría C, Grant WE. Projecting landscape changes in southern Chile: simulation of human and natural processes driving land transformation. Ecol Model 2001;222: 2841–2855.
10. Oyarzún C, Nahuelhual L, Núñez D. Los servicios ecosistémicos del bosque templado lluvioso: producción de agua y su valoración económica. Ambiente Desarro. 2005;20: 88–95.
11. Jaramillo A. Modelación del servicio ecosistémico de provisión y regulación hídrica bajo diferentes escenarios de uso de suelo: caso de estudio en el sur de Chile. Thesis, Magister en Ciencias Mención Recursos Hídricos, Facultad de Ciencias, Universidad Austral de Chile; 2004.
12. Armesto J J, León Lobos P, Kalin Arroyo M. Los bosques templados del sur de Chile y Argentina: una isla biogeográfica. In Armesto J, Villagrán C, Kalin Arroyo M, editors.

Ecología de los bosques nativos de Chile. Santiago de Chile, Editorial Universitaria, Universidad de Chile; 1996. pp. 23–28.

13. Villagrán C, Hinojosa LF. Historia de los bosques del sur de Sudamérica, II: Análisis fitogeográfico. Rev Chil Hist Nat. 1997:70: 1–267.

14. Barral MP, Maceira NO. Land-use planning based on ecosystem service assessment: A case study in the Southeast Pampas of Argentina. Agric Ecosyst Environ. 2012:154: 34–43.

15. León RJC. Vegetation In Soriano, A, editor. Río de la Plata Grasslands. In Coupland, RT. editor. Natural Grasslands. Elsevier, Amsterdam, NL; 1991. pp 380-338.

16. Viglizzo EF, Lértora F, Pordomingo AJ, Bernardos JN, Roberto ZE, Del Valle H. Ecological lessons and applications from one century of low external-input farming in the pampas of Argentina. Agric Ecosyst & Environ. 2001;83: 65-81.

17. Pengue WA. Transgenic Crops in Argentina: The Ecological and Social Debt. Bull Sci Technol Soc. 2005:25: 314–322.

18. Solbrig OT, Viglizzo E. Sustainable Farming in the Argentine Pampas: History, Society, Economy, and Ecology. David Rockefeller Center for Latin American Studies. Paper, (99/00), 1; 2000.

19. Viglizzo EF, Frank FC. Land-use options for Del Plata Basin in South America: Tradeoffs analysis based on ecosystem service provision. Ecol Econ. 2006;57: 140–151.

20. Laterra P, Nahuelhual L. Internalización de los servicios ecosistémicos en el ordenamiento territorial rural: bases conceptuales y metodológicas. In Paruelo JM, Jobbágy EG, Laterra P, Hernán Dieguez H, García Collazo , Panizza A, editors. Ordenamiento Territorial Rural. Conceptos, métodos y experiencias. INTA – UBA – FAO, Rome, Italy; 2015. pp. 86-106. At http://www.fao.org/3/a-i4195s.pdf Accessed July 2015.

21. Zelaya K. Descripción y modelización de la dinámica del uso de la tierra en la cuenca hidrológica de Mar Chiquita. Thesis Magister Scientiae. Facultad de Ciencias Agrarias, Universidad Nacional de Mar del Plata, Buenos Aires, Argentina; 2011..

22. Lima ML, Romanelli A, Massone HE. Assessing groundwater pollution hazard changes under different socio-economic and environmental scenarios in an agricultural watershed. Sci Total Environ. 2015:530: 333–346.

23. Verburg PH, Overmars KP. Combining top-down and bottom-up dynamics in land use modeling: Exploring the future of abandoned farmlands in Europe with the Dyna-CLUE model. Landsc Ecol. 2009:24: 1167–1181).

24. Hang S, Andriulo A, Sasal C, Nassetta MM, Portela S, Cañas AI. Integral study of atrazine behavior in field lysimeters in Argentinean humid pampas soils. Chil J Agricl Res. 2010;70: 104-112.

25. Sasal C, Andriulo A, Wilson M, Portela S. Pérdidas de Glifosato por Drenaje y Escurrimiento y Riesgo de Contaminación de Aguas. En: Camino M, Aparicio V (Eds) Aspectos Ambientales del Uso de Glifosato. Ediciones INTA, Buenos Aires, Argentina; 2010. pp. 103–114.

26. Booman GC, Calandroni M, Laterra P, Cabria F, Iribarne O, Vázquez P. Areal Changes of Lentic Water Bodies Within an Agricultural Basin of the Argentinean Pampas. Disentangling Land Management from Climatic Causes. Environ Manage. 2012:50: 1058-1067.

27. Nosetto MD, Paez RA, Ballesteros SI, Jobbágy EG. Higher water-table levels and flooding risk under grain vs. livestock production systems in the subhumid plains of the Pampas. Agric Ecosyst Environ. 2015:206: 60–70.

28. CIREN (Centro de Información de Recursos Naturales, CL) Estudio agrológico X Región. Descripciones de suelos, materiales y símbolos. Tomo I. Santiago, Chile. Centro de Investigación de Recursos Naturales; 2001. 199 pp.

29. Nahuelhual L, Carmona A, Lara A, Echeverría, C, González M. Land-cover change to forest plantations: Proximate causes and implications for the landscape in south-central Chile. Landscape Urban Plan. 2012;107: 12– 20.

30. Corporación Nacional Forestal. Monitoreo Nacional del Uso del Suelo en Chile, CONAF, Santiago, Chile; 2007.

31. Donoso Zegers, CSH, Spurr RS, Ambasht PM, Dansereau A, Sierra Pineda J Vázquez Soto DA et al. Las especies arbóreas de los bosques templados de Chile y Argentina autoecología Cartilla-Servicio Autónomo Forestal Venezolano (Venezuela) 4; 2006.

32. Salazar JC, Moscatelli GN, Cuenca MA, Ferrao RF, Godagnone RE, Grimberg HL et al. Carta de suelos de la provincia de Buenos Aires, Argentina. 1: 500.000 Instituto Nacional de Tecnología Agropecuaria (INTA), Buenos Aires, Argentina; 1988.
